# Author Correction: Nanoscale-femtosecond dielectric response of Mott insulators captured by two-color near-field ultrafast electron microscopy

**DOI:** 10.1038/s41467-021-22477-6

**Published:** 2021-04-01

**Authors:** Xuewen Fu, Francesco Barantani, Simone Gargiulo, Ivan Madan, Gabriele Berruto, Thomas LaGrange, Lei Jin, Junqiao Wu, Giovanni Maria Vanacore, Fabrizio Carbone, Yimei Zhu

**Affiliations:** 1 School of Physics, Ultrafast Electron Microscopy Laboratory, Nankai University, Tianjin, 300071 China; 2 Condensed Matter Physics and Material Science Department, Brookhaven National Laboratory, Upton, NY 11973 USA; 3 Institute of Physics, Laboratory for Ultrafast Microscopy and Electron Scattering (LUMES), École Polytechnique Fédérale de Lausanne, Station 6, Lausanne, 1015 Switzerland; 4 Department of Quantum Matter Physics, University of Geneva, 24 Quai Ernest-Ansermet, 1211 Geneva 4, Switzerland; 5 Department of Materials Science and Engineering, University of California, Berkeley, CA 94720 USA; 6 Department of Materials Science, University of Milano-Bicocca, Via Cozzi 55, 20121 Milano, Italy

**Keywords:** Phase transitions and critical phenomena, Nanowires, Transmission electron microscopy, Nanowires

Correction to: *Nature Communications* 10.1038/s41467-020-19636-6, published online 13 November 2020.

The original version of this Article contained errors in the captions of Figs. 2 and 4 and their corresponding main text. In the caption of Fig. 2, right panels should read right panel; middle panel should read right panel. In the caption of Fig. 4, *t*_2_ = 0 ps (coincidence between pump and probe, blue curve) and 1.28 ps (green curve) should read *t*_2_ = −0.4 ps (coincidence between pump and probe, blue curve) and 0.8 ps (green curve); *t*_2_ = 0 ps (blue curve) and 1.28 ps (green curve) should read *t*_2_ = −0.4 ps (blue curve) and 0.8 ps (green curve). In the main text of page 5, VO2 NWs should read VO_2_ NWs. In the main text of page 6, the blue box in the right panel should read the blue box in the middle panel; *t*_2_ = 0 ps and *t*_2_ = 1.28 ps should read *t*_2_ = −0.4 ps and *t*_2_ = 0.8 ps; decrease at 1.28 ps should read decrease at 0.8 ps; a time constant of ~145 fs should read a time constant of ~155 fs; a time constant of ~155 fs should read a time constant of ~240 fs.

This has been corrected in both the PDF and HTML versions of the Article.

