# Supplementary material for: Nanoscale-femtosecond dielectric response of Mott insulators captured by two-color near-field ultrafast electron microscopy
Source: Nat Commun. 2020 Nov 13;11:5770. doi: 10.1038/s41467-020-19636-6 (PMC7666229; doi:10.1038/s41467-020-19636-6)
Supplement: Supplementary file 2 — Description of Additional Supplementary Files [file 41467_2020_19636_MOESM2_ESM.pdf]

Description of Additional Supplementary Files

Supplementary Movie 1: Temporal elvolution of one-colour PINEM images of a single VO_2 NW with P1 optical pulse (duration of 50 fs, $\lambda = 800$ nm, fluence of ~4.1 mJ/cm^2) polarized perpendicularly to the NW axis.
